# Asymptotics for metamaterials and photonic crystals

**DOI:** 10.1098/rspa.2012.0533

**Published:** 2013-04-08

**Authors:** T. Antonakakis, R. V. Craster, S. Guenneau

**Affiliations:** 1 Department of Mathematics, Imperial College London, London SW7 2AZ, UK; 2 European Organization for Nuclear Research, CERN 1211, Geneva 23, Switzerland; 3 Fresnel Institute, UMR CNRS 7249, Aix-Marseille University, Marseille, France

**Keywords:** metamaterials, cloaking, negative refraction, homogenization

## Abstract

Metamaterial and photonic crystal structures are central to modern optics and are typically created from multiple elementary repeating cells. We demonstrate how one replaces such structures asymptotically by a continuum, and therefore by a set of equations, that captures the behaviour of potentially high-frequency waves propagating through a periodic medium. The high-frequency homogenization that we use recovers the classical homogenization coefficients in the low-frequency long-wavelength limit. The theory is specifically developed in electromagnetics for two-dimensional square lattices where every cell contains an arbitrary hole with Neumann boundary conditions at its surface and implemented numerically for cylinders and split-ring resonators. Illustrative numerical examples include lensing via all-angle negative refraction, as well as omni-directive antenna, endoscope and cloaking effects. We also highlight the importance of choosing the correct Brillouin zone and the potential of missing interesting physical effects depending upon the path chosen.

## 1. Introduction

Photonic crystal (PC) media [1,2] and metamaterials [3] are topical areas in optics, and both involve non-resonant or resonant interactions created by waves within periodic structures. Such structures are of considerable current interest [4], with applications to invisibility and cloaking [5], among others. In photonics, a typical structure may involve multiple cylindrical holes [6], and in metamaterials, the peculiar properties of split-ring resonators (SRRs) [7] are typical building blocks. In both cases, the physics is neatly encapsulated and displayed by dispersion diagrams that relate the Bloch wavenumber in the irreducible Brillouin zone to the frequency; these illustrate essential features such as band gaps of frequencies where propagation in an infinite periodic structure is disallowed. Such dispersion diagrams for infinite periodic media can then be used to design the size and geometry of structural elements within a cell to create particular optical features. We turn to arrays of cylindrical holes and to SRRs in order to illustrate the versatility of a new technique that creates effective homogenized models, even at high frequencies, and in doing so also uncover details of how the SRR geometry affects the metamaterial properties. In this article, we treat a specific polarization in electromagnetism, transverse electric (TE), whereby the magnetic field is perpendicular to the plane of periodicity, and we consider perfectly conducting structural elements, such that the holes have a Neumann condition upon them; this is of particular interest as there has been discussion that homogenization theory is invalid for this case.

**Figure 1.**
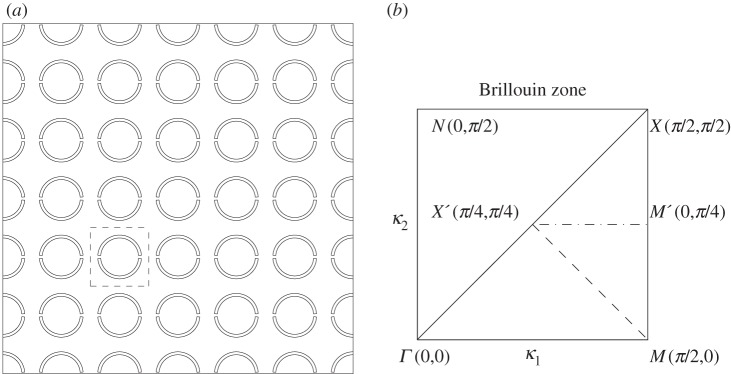
(*a*) An infinite square array of SRRs with the elementary cell shown as the dashed line inner square. (*b*) The irreducible Brillouin zone, in wavenumber space, used for square arrays in perfectly periodic media based around the elementary cell shown of length 2*l* (with *l*=1).

Many materials of abiding interest in physics are created from periodically repeating cells such as those of figure 1*a*, which shows a square array of SRRs with a square cell as the dashed square. Given such a medium, with, say, a defect or many hundreds of cells within a macrocell, it is attractive to replace it with an effective medium on a macroscale; naturally, one hopes that the effective replacement continuum model material captures the behaviour created by the microscale. For long waves, in the quasi-static low-frequency limit, there is an established theory, homogenization theory [4,8–11], that replaces a microstructured medium with an averaged macroscale model; this is highly successful and an attractive approach for low-frequency waves with wavelengths many times the typical cell size. However, many of the features of interest in real PCs, or other periodic structures, such as all-angle negative refraction (AANR) [12,13] or ultra-refraction [14] occur at high frequencies where the wavelength and microstructure dimension are of similar orders. Therefore, the conventional low-frequency classical homogenization clearly fails to capture the essential physics, and a different approach to distill the physics into an effective model is required. Fortunately, a high-frequency homogenization (HFH) theory, developed in Craster *et al.* [15], is capable of capturing features such as AANR and ultra-refraction for some model structures [16]. Somewhat tangentially, there is existing literature in the analysis community on Bloch homogenization [17,18] and, in particular, Hoefer & Weinstein [19], which is related to what we call HFH. There is also literature on developing homogenized elastic media, with frequency-dependant effective parameters, based upon periodic media [20]. There is therefore considerable interest in creating effective continuum models of microstructured media that break free from the conventional low-frequency homogenization limitations. In this article, we turn our attention to microstructures of abiding interest, perfectly conducting holes (in TE polarization) that have been much studied in the literature [21] and to the topical SRR metamaterial structure [7,22]; for both cases, we illustrate how the general HFH model is used and the asymptotic behaviour of the dispersion curves found. Interesting details such as the behaviour at crossing points, and degenerate behaviour, where the local behaviour in the dispersion curve switches from quadratic to linear are all found from effective macroscale equations that have the microscale completely captured within coefficients. In the SRR case, the homogenization procedure must be performed numerically, and this is done here; hence, with minor modifications, the methodology can now be applied to any geometry within a cell.

On the topic of quasi-static homogenization, a series of papers [23–25] draws perplexing conclusions about the possibility of using homogenization at all, even for the lowest acoustic band in the dispersion diagram. For doubly periodic perfectly conducting inclusions with Dirichlet (transverse magnetic (TM) polarization, i.e. with an electric field perpendicular to the plane of periodicity) boundary conditions, there is no intercept of the acoustic branch with the origin, and conventional homogenization fails; there is no controversy with this deduction. Notably, the HFH has no such failing, as shown in Craster *et al.* [15]. The discrepancy in the literature is with (cylindrical) perfectly conducting inclusions with Neumann (TE polarization) boundary conditions for which analytical multi-pole methods [24] produce linear asymptotics as the acoustic branch approaches zero wavenumber and the precise behaviour is not replicated by conventional homogenization; the slope of the asymptotics is important as it is related to the effective refractive index of the medium for long waves. Furthermore, if the cylinders have finite dielectric properties, then the limits of zero conduction and of zero wavenumber do not commute. Complementary to this are claims that homogenization theory will actually operate correctly, see the comments and responses by Halevi *et al.* [26] and Nicorovici *et al.* [27]. Here, we advance the theory of homogenization to higher frequencies and, as a corollary, are able to demonstrate conclusively, using our approach, that one can indeed homogenize perfectly conducting cylinders in the TE polarization for the quasi-static low-frequency limit.

Parallel to the electromagnetic setting is a mathematically identical interest in, mainly cylindrical, periodic inclusions in acoustics, water waves and anti-plane elasticity. Homogenization in those settings for long waves relative to the cell spacing leads to effective equations [28,29] that do not appear to have any issues, and if, furthermore, the inclusions are taken to be small, then singular perturbation theory can be employed [30], or asymptotic coupled mode theory [31], to good effect.

We generate an HFH theory for repeating cells containing Neumann inclusions; this limiting case is not covered within Craster *et al.* [15] and is of independent interest, even at low frequencies. The general high-frequency model is developed in §2, with the low-frequency limit covered in a §2*b*. Armed with the general theory, we verify its efficacy upon the well-studied cylindrical inclusion case (§3*a*), which allows us to demonstrate that homogenization does indeed work despite arguments to the contrary, and for all frequencies, not just in the low-frequency limit. The cylindrical inclusions contrast with the SRRs, where additional branches split from those in the cylindrical case, and for which interesting asymptotic results emerge in §3*b*. Section 4 gives an overview of complementary geometrical asymptotic limits. The HFH asymptotics give additional physical insight that then motivates us to explore further features of the SRRs and cylinders in §5. Finally, concluding comments and remarks are presented in §6.

## 2. General theory

For infinite perfectly periodic media, consisting of elementary cells that repeat, one focuses attention on a single elementary cell; quasi-periodic Floquet–Bloch boundary conditions describe the phase shift as a wave moves through the material, and dispersion relations then relate the Bloch wavenumber, the phase shift, to frequency. The Bloch wavenumber is a vector, and figure 1*b* shows the irreducible Brillouin zone [32] *ΓXM* associated with a single repeating elementary square cell containing, say, a circular hole, also shown is the smaller triangle for a group of four repeating cells. The dispersion diagrams we show are frequency versus wavenumber around the edges of the Brillouin zone, as is traditional in solid-state physics. There are occasions when doing this misses interesting details [33], and we illustrate this later by noting that a perfectly flat path occurs for a square array of strings along *MX*^′^ (a path that is missed by going around the path *ΓXM*) and that an almost flat band occurs for an array of cylinders; this flat band leads to directional standing wave patterns. We also note that the symmetry of the hole is important, and for the two thin ligament SRR of figure 1*a*, one should use the square *ΓMXN*.

The eigensolutions that emerge are the Bloch modes at the edges of the Brillouin zone, and when these eigensolutions are perfectly in phase or out of phase across the cell, then standing waves exist, and there are standing wave frequencies (whose frequencies can be high). Asymptotic techniques based around high-frequency long-wave asymptotics have recently been developed [15], and Schrödinger ordinary differential equations in one-dimensional periodic media (or partial differential equations in two dimensions) emerge; this approach also works for microstructured discrete [34] or frame-like media [35]. These recent theories avoid the issue of perfectly conducting holes and have material properties varying periodically on the scale of the elementary cell and only treat model problems, mainly in one dimension, for which completely analytic progress can be made. The key idea for periodic media is to replace the complicated microstructured medium with an equivalent, effective, continuum on a macroscale, that is, one wishes to homogenize the medium, even when the wavelength and microstructure may be of similar scales.

The theory is ultimately not limited to just reproducing dispersion curves asymptotically, and it can be adjusted to treat localized defect modes and other features due to local non-periodic material changes or boundaries, with these effects coming through in extra forcing terms within the continuum partial differential equations.

We begin with a two-dimensional structure composed of a square lattice geometry of identical cells with identical holes inside each of them. The side length of the direct lattice base vectors, i.e. the side of each square cell, is taken as 2*l*. Note that for simplicity, equal length lattice vectors and a square lattice are assumed, and both assumptions could be relaxed. These elementary cells define a length scale that is the microscale of the structure. As noted above, real structures could be created from many hundreds or thousands of such elementary cells, and we introduce a macroscale length denoted by *L* that could be viewed as a characteristic overall dimension of the structure. The ratio of these scales, *ϵ*≡*l*/*L*, is assumed small.

Each cell is identical in geometry and the material within each cell is characterized by two periodic functions, in ***ξ***≡(*x*_1_/*l*,*x*_2_/*l*), namely 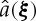 and 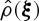. Depending on the application, these could be stiffnesses and density for shear horizontal polarized elastic waves or inverse permittivity and permeability in electromagnetism. The geometry is specific in the sense that it contains an arbitrary hole, or set of holes, and boundary conditions have to be prescribed on the hole. In this study, Neumann conditions will be used that are the natural boundary conditions for perfectly conducting holes in the transverse TE polarization of electromagnetism or for stress-free holes in anti-plane shear elasticity.

A time-harmonic dependence of propagation 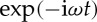, with frequency *ω*, is assumed throughout, and henceforth suppressed, and a non-dimensionalization by setting 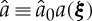 and 
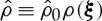
, where 
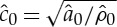
 is the characteristic wave speed, leads to the resulting equation of study,
2.1
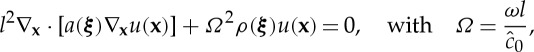
on 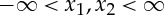; *Ω* is the non-dimensional frequency and *u* is the out-of-plane displacement in elasticity or the *H*_3_ component of the magnetic field in TE polarization.

The two-scale nature of the problem is incorporated using the small and large length scales to define two new independent coordinates, namely **X**=**x**/*L*, and ***ξ***=**x**/*l*. Equation (2.1) then becomes
2.2
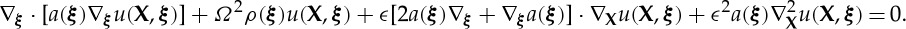
Standing waves occur when there are periodic (or anti-periodic) boundary conditions across the elementary cell (in the ***ξ*** coordinates), and these standing waves encode the local information about the multiple scattering that occurs by the neighbouring cells. The asymptotic technique is then a perturbation about these standing wave solutions, as these are associated with periodic and anti-periodic boundary conditions, which are, respectively, in-phase and out-of-phase waves across the cell; the conditions in ***ξ*** on the edges of the cell, ∂*S*_1_, are known,
2.3
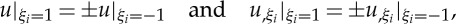
with the + or − for periodic or anti-periodic cases, respectively. We now pose an ansatz for the field and the frequency,
2.4
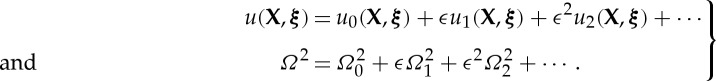
The *u*_*i*_(**X**,***ξ***)'s adopt the boundary conditions (2.3) on the edge of the cell. An ordered set of equations emerge indexed with their respective power of *ϵ*, and are treated in turn,
2.5
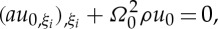

2.6
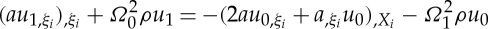

and
2.7
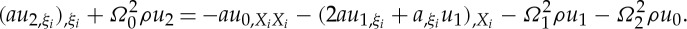
The leading order equation (2.5) is independent of the long scale **X** and is a standing wave on the elementary cell excited at a specific eigenfrequency *Ω*_0_ and associated eigenmode *U*_0_(***ξ***;*Ω*_0_), modulated by a long-scale function *f*_0_(**X**) and so
2.8
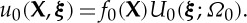
At this point, we will assume isolated eigenfrequencies, but repeated eigenvalues arise and are discussed later. The entire aim is to arrive at a partial differential equation for *f*_0_ posed entirely upon the long scale, but with the microscale incorporated through coefficients that are integrated, not necessarily averaged, quantities.

Before we continue to the next order, equation (2.6), we define the Neumann boundary conditions on the holes, ∂*S*_2_,
2.9
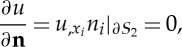
where **n** is the unit outward normal to ∂*S*_2_, and which in terms of the two scales and *u*_*i*_(**X**,***ξ***) become
2.10
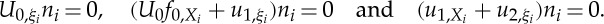
The leading order eigenfunction *U*_0_(***ξ***;*Ω*_0_) must satisfy the first of these conditions. Moving to the first-order equation (2.6), we invoke a solvability condition by integrating over the cell the product of equation (2.6) and *U*_0_ minus the product of equation (2.5) and *u*_1_/*f*_0_(**X**). The eigenvalue *Ω*_1_ is zero, and we can solve for *u*_1_=*f*_0,*X*_*i*__*U*_1_*i*__(***ξ***), so **U**_**1**_ is a vector field. By re-invoking a similar solvability condition for equation (2.7), we obtain the desired partial differential equation for *f*_0_
2.11
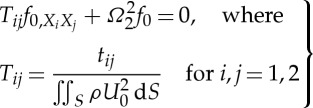
posed entirely on the long scale **X**. The tensor *t*_*ij*_ consists of integrals over the microcell in ***ξ*** and is ultimately independent of ***ξ***. The formulations for *t*_*ij*_ read
2.12
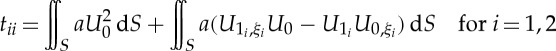
and
2.13
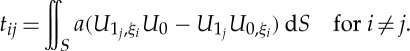
There is no summation over repeated suffices for the *t*_*ii*_. Notably, although the general approach follows Craster *et al.* [15], there are subtle differences induced by the Neumann conditions; the solution there for the first-order equation is of the form **U**_**1**_(***ξ***;*Ω*_0_)=**V**_**1**_(***ξ***;*Ω*_0_)−***ξ****U*_0_(***ξ***;*Ω*_0_). We no longer use the auxiliary function **V**_**1**_(***ξ***;*Ω*_0_), which turns out to be numerically awkward. Instead, solving directly for *U*_1_*i*__ is much simpler. *U*_1_*i*__ is a solution of the non-homogeneous partial differential equation, 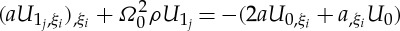, with the same boundary conditions as the leading order equation on ∂*S*_1_ and with the second boundary conditions of (2.10) on ∂*S*_2_. The numerical solutions of *U*_0_, and subsequently *U*_1_*j*__, are computed using a standard finite-element package [36], thereby allowing us to treat general geometries.

### (a) Repeated eigenvalues

A potential limit case not treated above is that, for some standing wave frequencies, there is more than one propagating mode, i.e. there are repeated eigenfrequencies with multiplicity *p*. The general solution to the leading order problem then becomes
2.14
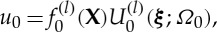
with summation assumed over repeated superscripts *l*, and
2.15
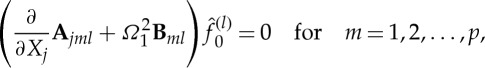
with *Ω*_1_ not necessarily zero, and
2.16
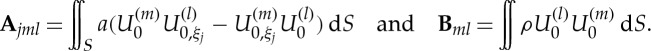
The coupled system of partial differential equations (2.15) for the 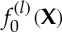 are solved, but become degenerate if *Ω*_1_=0. For most of the examples treated in this article, *Ω*_1_ is found to be zero. We then have to proceed in a similar way used to obtain equation (2.11). We get another degenerate case where the coupled partial differential equations become
2.17
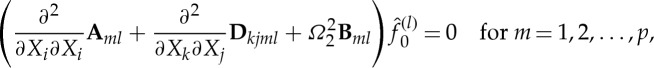
with the following coefficients:
2.18
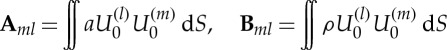
and
2.19
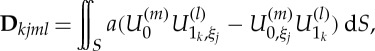
and the range of variation of *l* is equal to the multiplicity of the eigenvalue.

### (b) The classical long-wave zero-frequency limit

The current theory simplifies if one enters the classical long-wave, low-frequency limit where *Ω*^2^∼*O*(*ϵ*^2^) as *U*_0_ becomes uniform, and without loss of generality is set to be unity, over the elementary cell. The final equation is again (2.11) where the tensor *t*_*ij*_ simplifies to
2.20
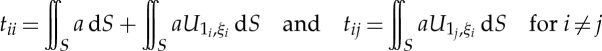
(with no summation over repeated suffices) and 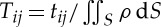. In the above equations, *U*_1_*i*__ is a solution of
2.21
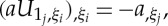
with boundary conditions (*f*_0,*X*_*i*__+*u*_1,*ξ*_*i*__)*n*_*i*_=0 on the hole boundary. In the illustrative examples of circles or SRRs in an otherwise homogeneous medium, *a* is constant so equation (2.21) is the same as that for *U*_0_, but with different boundary conditions. The specific boundary conditions for *U*_1_*j*__ are
2.22
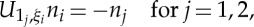
where *n*_*i*_ represents the normal vector components. The role of **U**_**1**_ is to ensure Neumann boundary conditions hold and the tensor contains simple averages of the stiffness and density (equivalently inverse permittivity and permeability for TE modes) supplemented by the correction term that takes into account the boundary conditions at ∂*S*_2_. Equation (2.20) is the classical expression for the homogenized coefficient in a scalar wave equation with periodic coefficient *a*; (2.21) is the well-known annex problem of electrostatic type set on a periodic cell [8,9] and also holds for the homogenized vector Maxwell system, where **U**_**1**_ now has three components and *i*,*j*=1,2,3 [37].

## 3. Illustrative examples

We now illustrate the theory using arrays of circular holes and SRRs and for perfect structures for which full dispersion diagrams can be found numerically. To compare with the general theory, we now specialize in Bloch waves where 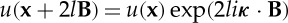, which translates in two-scale coordinates as 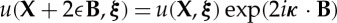, where **B** is either **b**_1_, **b**_2_ or **b**_1_+**b**_2_ with **b**_*i*_ as the orthonormal unit vectors. Floquet–Bloch boundary conditions on the cell imply 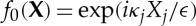. In this notation, *κ*_*j*_=*K*_*j*_−*d*_*j*_ and *d*_*j*_=0,*π*/2,−*π*/2 depending on the location in the Brillouin zone. Equation (2.11) and the frequency expansion of equation (2.4) lead to
3.1
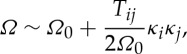
with similar results for (2.17); thus, one can compare directly with the full numerics. It is worth while noting the use of *T*_*ij*_ coefficients, as their sign and absolute value give information about the group velocity for the specified frequencies and locations of the Brillouin zone.

### (a) Lattice of square cells with circular inclusions

**Figure 2.**
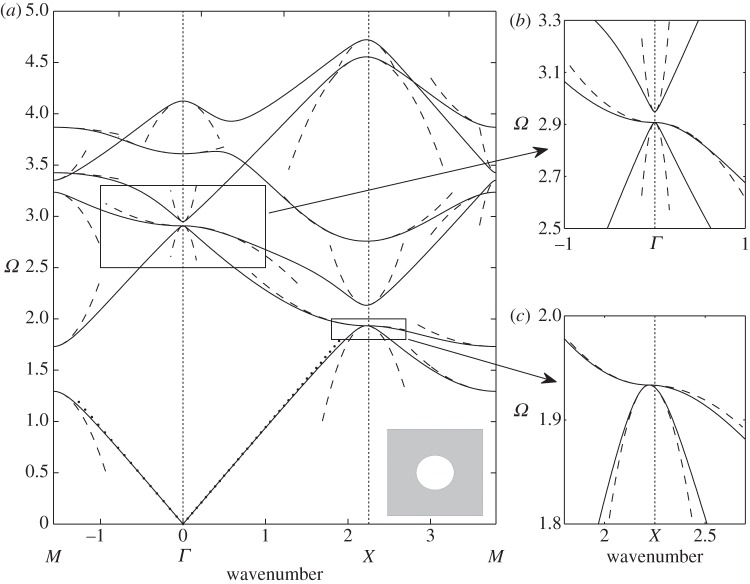
The dispersion diagram for an array of square cells (side 2) with circular inclusions of radius 0.4. (*a*) Dispersion curves from numerics (solid lines), the asymptotic solutions from HFH theory (dashed lines) and the linear long-wave classical homogenization asymptotics are the dotted lines emerging from the origin. (*b*,*c*) Enlargements near repeated eigenvalues where the asymptotics from (2.17) are used.

**Figure 3.**
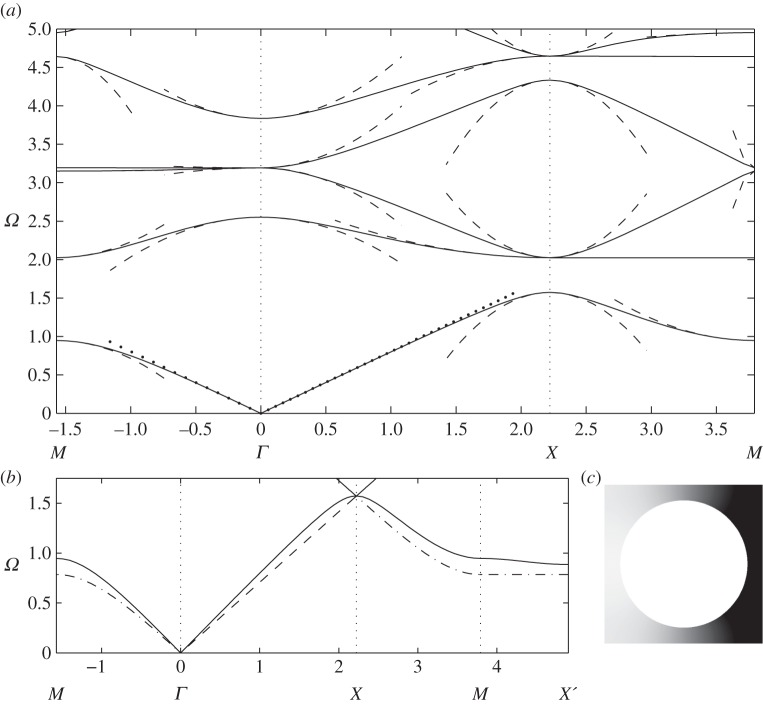
The dispersion diagram is shown for square cells (of side 2) with circular inclusions of radius 0.8. (*a*) Follows figure 2*a*, but for this larger radius. (*b*) The acoustic branch (solid lines) with the dispersion curves from a frame of thin strings also shown (dashed lines). (*c*) The eigensolution at *M* on the acoustic branch that is in phase vertically and out of phase horizontally with the variation concentrated along the horizontal pieces.

The dispersion curves for arrays of cylindrical holes have been treated by many authors [38] among others, and we proceed by computing them numerically using COMSOL Multiphysics and by using the asymptotics developed in §2. We choose to illustrate them for two hole radii, with the geometry as a square of side length 2 and the inclusion's radius is either 0.4 or 0.8, in figures 2 and 3, respectively around the edges of the irreducible Brillouin zone of figure 1*b*. These dispersion diagrams illustrate interesting features such as a Bragg stop band (due to multiple scattering of light between the circular inclusions) that is absent for the small holes (figure 2), but which develops for larger holes (figure 3) as the multiple scattering becomes more pronounced. In both figures 2 and 3, it is clear that the asymptotics capture the fine details of the dispersion curves near each standing wave frequency. The classical long-wave limit near the origin has a linear asymptotic dispersion curve, from §2*b*, and these capture the gradient of numerically calculated paths near the origin perfectly in both figures, showing that homogenization can indeed be used in TE polarization. Figure 2*b*,*c* shows expanded regions near repeated roots using the asymptotics of §2*a* with the theory capturing fine details such as changes in curvature; notably, all of this behaviour is encapsulated in the tensor *T*_*ij*_ that is independent of the microscale coordinates.

As the hole radius increases, a stop band opens up with the acoustic branch isolated from the others (figure 3*a*), and the changes in the field *u* are concentrated along the relatively thin pieces of remaining material; a typical eigensolution is shown in figure 3*c*. This motivates one to replace the circular array by an array of simple strings or thin ligaments in the form of a square frame, for which the dispersion relation
3.2
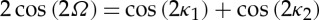
is easily found [35,39]. This dispersion relation, for the acoustic branch, is shown in figure 3*b* and is a good approximation to that of large holes capturing the main features. If one considers the path *MX*^′^ in the Brillouin zone, one notices that (3.2) gives a completely flat band; this is related to the striking occurrence of directional standing waves [33,40–42] that are of current interest in discrete or frame-like structures. Notably, the continuum system of cylinders shares this feature, although the path is no longer perfectly flat, suggesting that directional standing waves forming cross-like vibrations will exist here also; this is explored in §5.

### (b) Split-ring resonator

**Figure 4.**
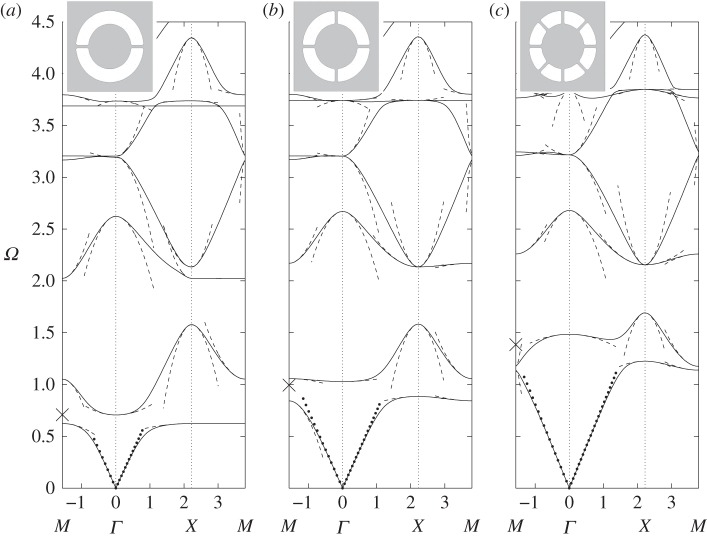
The dispersion diagram shown for an array of square cells (side 2) containing circular holes of radius 0.8, and with circular inclusions of radius 0.5, attached to the rest of the cell by two, four and eight thin ligaments in (*a*–*c*), respectively. The dispersion curves from numerics are shown as solid lines, the asymptotic HFH results are shown as dashed lines, with the low-frequency linear classical homogenization shown dotted emerging from the origin. The thin ligament approach of §4*a* gives estimates for the first non-zero eigenvalue at *Γ*, which are the crosses on the frequency axis. Numerical values for these estimates are 0.7082, 0.9941, 1.3851 versus finite-element simulations of 0.7058, 1.0281, 1.4845 for two, four and eight ligaments, respectively. The sixth mode of figure 4*b* is flat at frequency 3.741 and corresponds to the dipole mode described in §4 with approximate frequency 3.6824.

We now modify the simple circular hole by inserting a smaller circular inclusion within it attached to the hole's walls by ligaments. The ratio of width to length of the ligaments used, as well as their number, play a major role in the underlying physics. Again, there have been many numerical studies for dispersion diagrams, for instance in Guenneau *et al.* [43], and semi-analytical work for narrow gaps as in Llewellyn-Smith & Davis *et al.* [44]. Figure 4*a*–*c* shows the dispersion curves together with the asymptotics obtained from §4*a* for ‘long’ ligaments with a width to length ratio *η*=*h*/*l* of 0.2. The new feature in comparison with figure 3 is the appearance of a low-frequency stop band below the Bragg stop band, whose upper edge remains virtually unaffected by the insertion of the resonator in each circular inclusion of the array. The low-frequency stop band is associated with a localized mode upon resonance of the resonator, and is responsible for artificial magnetism in metamaterials in TE polarization. Physically, an array of cylinders with capacitive splits such as in figure 4*a*–*c* respond resonantly to radiation with the magnetic field when it is oriented along the cylindrical axes [22]. The oscillating magnetic field induces currents to run around the perfectly conducting rings. These currents feel a finite inductive impedance due to the finite size of the conducting loops, while they feel a capacitive impedance due to the capacitive gaps within the conducting loops. This gives rise to a resonant response of the system where the resonance is driven by the magnetic field of the radiation with a consequent resonant enhancement of the magnetic polarizability of each cylinder. If the array period is sufficiently small compared to the wavelength of the applied magnetic field, then the metamaterial is described by an effective magnetic permeability [7]. This effective magnetic permeability displays a strong dispersion near the resonance frequency of the SRR, and it can become negative in the frequency band just above the resonance frequency, i.e. in the low-frequency stop band. Sadly, the low-frequency stop band appears at frequencies already beyond the scope of classical homogenization, but fortunately HFH captures its finer details, as in figure 4*a*–*c*, and thus unveils the fascinating physics of artificial magnetism. For instance, the inverted curvature of the second dispersion curve around the *Γ* point in figure 4*a*, which is a hallmark of a Mie resonance driving the artificial magnetism [22] is captured by the HFH, as is the flat band along the *XM* path, which is associated with a localized mode in the SRR (which is therefore insensitive to any variation of the Floquet–Bloch phase shift across the unit cell along this path). The highly dispersive physics of the low-frequency stop band will reveal the ultra-refraction and AANR effects shown in §5.

### (c) A thin annulus with holes

**Figure 5.**
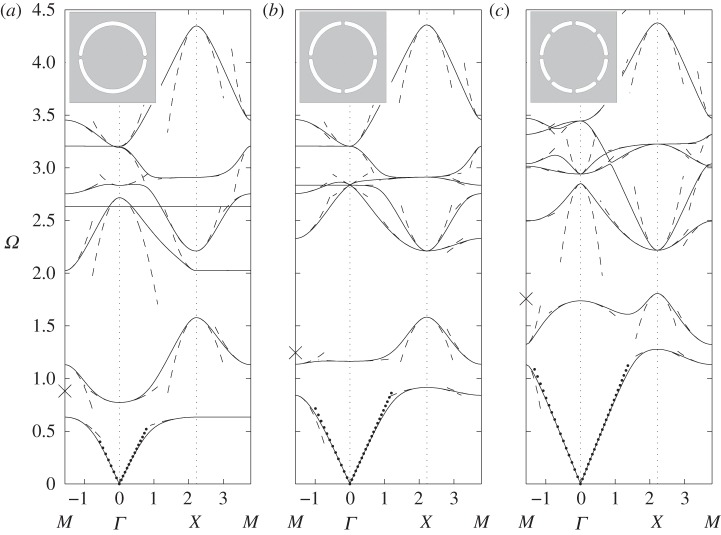
The dispersion diagrams, same as figure 4, showing two, four and eight ligaments in (*a*–*c*), respectively, but with the inclusion radius now 0.7. The resonant fourth mode is almost flat in (*a*) with the dipole estimate giving a frequency of 2.6303. The crosses on the frequency axis in (*a*–*c*) show the estimates (0.88176, 1.24539 and 1.75670, respectively) from (4.3) for the resonant frequency at *Γ* and the finite-element simulations give 0.7726, 1.1636 and 1.7378.

For contrast, we investigate the effect of shortening the ligaments so *h*/*l* is of order 1; adding more cuts in the thin annulus preserves the lowest resonant frequency, but it becomes less sharp, which makes the low-frequency band gap wider. A physical side effect is that artificial magnetism weakens when the resonance is less sharp, i.e. effective permeability is less dispersive and might not reach large enough negative values for potential metamaterial applications, such as lensing via negative refraction. On the other hand, the metamaterial might work over a broader range of frequencies if the stop band widens, and this could be a design requirement. This leads to a subtle balance between having a sharp resonance and a wide low-frequency stop band; HFH can provide useful guidance towards achieving such a goal. Figure 5*a*–*c* shows the dispersion curves and asymptotics for, respectively, two, four and eight short ligaments. The inverted curvature of the second dispersion curve around *Γ* point flattens for four holes (hence, the Mie resonance responsible for artificial magnetism fades away), and the curvature actually changes sign when comparing two and eight ligaments. This illustrates the fact that it is not enough to use simple models (such as electrical circuits) to fully grasp the physics of SRRs. Indeed, models such as those of §4 merely provide frequency estimates for the resonance occurrences, but cannot actually reproduce asymptotically the dispersion curves as HFH does.

**Figure 6.**
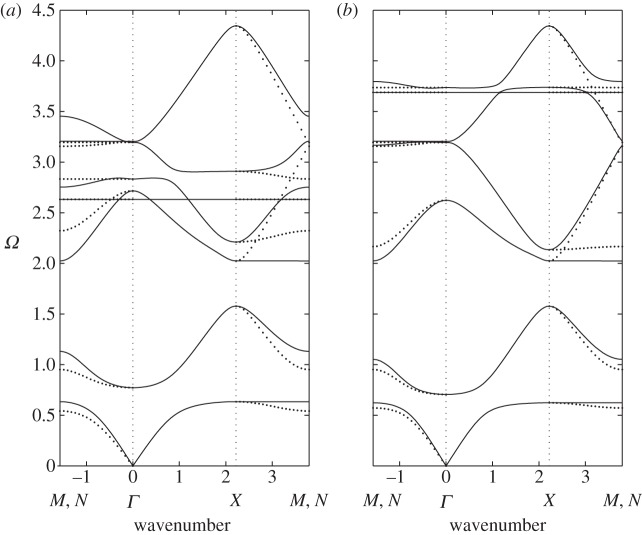
The dispersion diagrams for the (*a*) short and (*b*) thin two-ligament SRR illustrating the changes induced by using triangle *ΓXM* (solid lines) and *ΓXN* (dotted lines). Notably for two ligaments, the symmetries are that the irreducible Brillouin zone is actually the square *ΓNXM* of figure 1*b*.

A note of warning is worth sounding regarding the irreducible Brillouin zone: it is all too easy to overlook the fact that the two-ligament SRR does not have the appropriate symmetries such that one can use just the triangle *ΓXM* as the irreducible Brillouin zone. Instead, one should use *ΓNXM*, and to highlight this, we show in figure 6 the dispersion curves using different triangular paths, namely *ΓXM* and *ΓXN*. At first sight, the differences are not substantial, but closer inspection at higher frequencies shows that one could incorrectly find complete band gaps where there are partial gaps (figure 6*a*).

## 4. Geometric asymptotics

**Figure 7.**
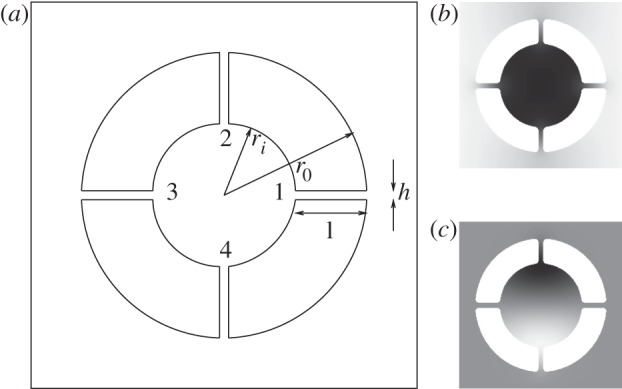
The thin ligament geometry for four ligaments, in (*a*), with *h*/*l*≪1 and the ligaments numbered as in the text. (*b*) The eigenfunction at *Γ* for the first non-zero eigenvalue (1.0281) of figure 4*b* showing the central region moving as a rigid body and the variation localized along the thin ligaments. (*c*) The dipole mode at a frequency of 3.741442 for the sixth mode of figure 4*b*. A simple approximation of a cavity dipole 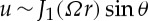 gives the estimate 3.6824.

It is clear that there are geometrical approximations that can be used, mainly for the acoustic or other low-frequency branches, where the inner cylinder for the SRR acts as a resonator or an effective mass, or where the cylinders are large and the walls separating them are thin. We briefly treat these theories here as they are complementary to the technique we have developed and allow for additional insight. Resonances do not only occur at low frequencies, in figure 4*a*–*c*, higher resonances are also clearly visible with the flat sixth mode being particularly noticeable; this is the dipole mode of figure 7*c*.

### (a) Thin ligaments for split-ring resonators

As noted by Movchan & Guenneau [45], one can take advantage of the thin ligaments when *h*/*l*≪1 to obtain asymptotic estimates for the lowest eigenvalues of multi-structures as in Kozlov *et al.* [46]. The current example is of interest as the inner cylindrical mass oscillates as a rigid body being connected to the outer medium via the thin ligaments, which act as simple strings. An illustration of this using four ligaments is shown in figure 4*b* alongside a sketch of the system. The outer medium is either stationary or oscillates as a rigid body, as this is the case of the first mode at point *M* or the second mode at point *Γ* of the irreducible Brillouin zone in figure 1*b*. Assuming an even number of ligaments, *n*, each placed opposite another, one can arrive at an asymptotic model similar to that of Movchan & Guenneau [45]. Taking the inner and outer radii as *r*_*i*_ and *r*_0_, respectively, with each ligament of width *h*_*j*_, we define *x*_*j*_ (for *j*=1…*n*) as a local coordinate along each ligament with *x*_*j*_=0 at the inner radius and *x*_*j*_=*r*_0_−*r*_*i*_=*l* at the outer radius, and for clarity, we number the ligaments anti-clockwise as in figure 7. In this low-frequency limit, the inner cylinder moves as a rigid body with displacement *u*_0_, and one simply solves *n* coupled string equations
4.1
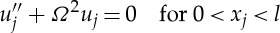
for the displacement *u*_*j*_(*x*_*j*_) and *j*=1…*n*. The boundary conditions are that *u*_*j*_(*l*)=0 and *u*_*j*_(0)=*u*_0_, and the rigid body motion of the mass *M* induces a jump condition
4.2
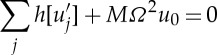
at the origin, where the sum is over the first *n*/2 strings and [⋅] denotes the jump in the derivative between each string and the string that is placed opposite to it. The upshot is that a simple dispersion relation emerges as
4.3
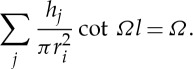
The frequencies predicted from (4.3) are shown as crosses in figures 4*a*–*c* and 5*a*–*c*, with the numerical and asymptotic values given in each figure caption.

## 5. Applications

We now illustrate the theory alongside applications to lensing, cloaking and endoscope effects in PCs and metamaterials. Electric line sources will be used to observe the anisotropic effects. These sources are in the direction perpendicular to the paper plane as if the geometries in question were infinite in depth (fibres). Indeed, infinite long cylinders or SRRs that are perfect magnetic conductors are subject to Neumann-type boundary conditions when solving for TE-polarized waves.

### (a) All-angle negative refraction in perfect conducting photonic crystals

**Figure 8.**
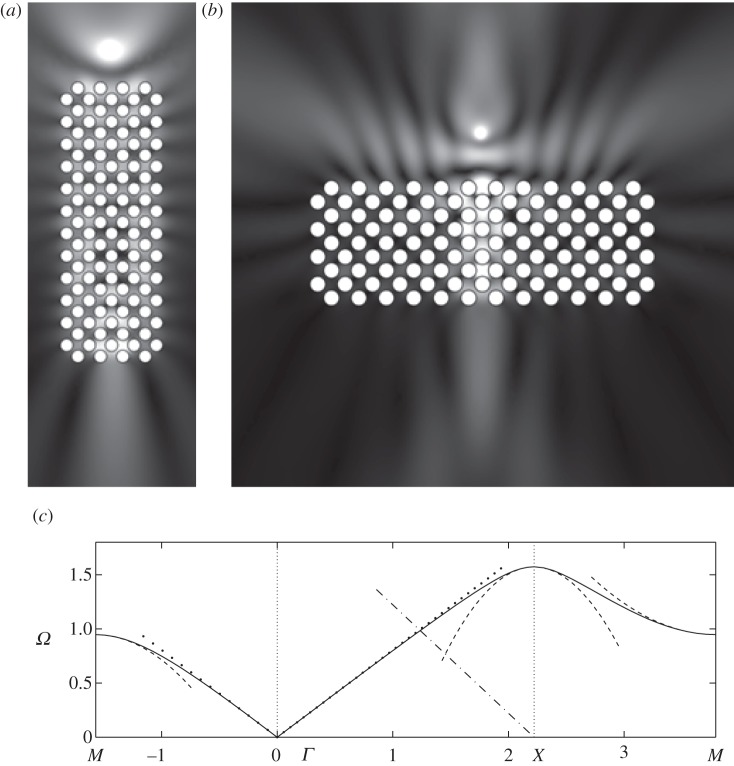
Endoscope and flat lens with perfect conducting PCs: (*a*) a line source at frequency *Ω*=1.564 located above a rectangular PC consisting of 112 perfect conducting circular inclusions as in figure 3 leads to a ‘photonic jet’ effect below (endoscope effect); (*b*) a line source at frequency *Ω*=1.125 located above a rectangular PC consisting of 112 perfect conducting circular inclusions as in figure 3 leads to an elongated image underneath (Veselago lens); (*c*) zoom on dispersion diagram of figure 3. Note that each cell in the arrays in (*a*) and (*b*) has been rotated through an angle *π*/4.

**Figure 9.**
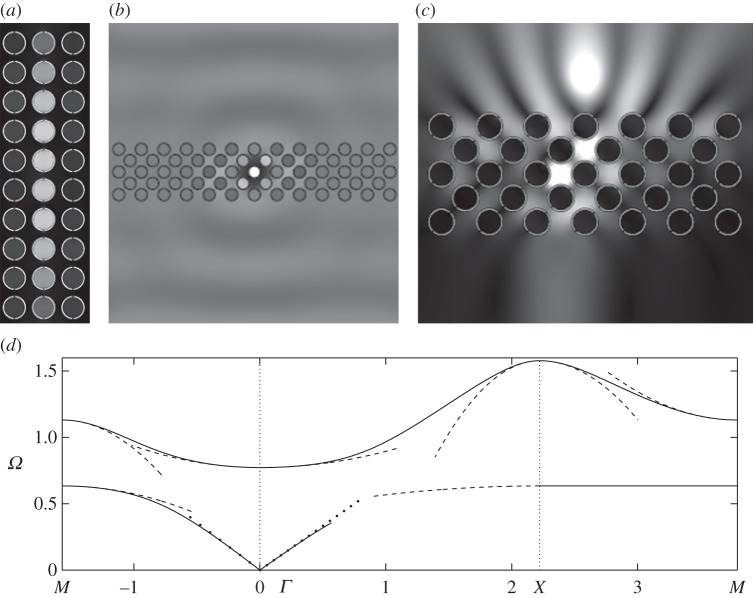
Line defect, concentration and endoscope effects in square arrays of SRRs with two holes: (*a*) a line source at frequency *Ω*=0.6 located in the centre of a rectangular metamaterial consisting of 30 SRRs as in figure 5*a* produces a wave pattern of the line defect type; (*b*) a line source at frequency *Ω*=0.78 located in the centre of a rectangular metamaterial consisting of 33 SRRs shaped as in figure 5*a* produces a plane wave outside the array (omni-directive antenna via ultra-refraction); (*c*) a line source at frequency *Ω*=1.2 located above a rectangular metamaterial consisting of 33 SRRs shaped as in figure 5*a* leads to a concentration effect underneath; (*d*) zoom on dispersion diagram of figure 5*a*. Note that each cell in the arrays of SRRs in (*b*) and (*c*) is tilted by an angle *π*/4, unlike for (*a*).

One of the most topical subjects in photonics is the so-called AANR, which was first described in Zengerle [13]. AANR allows one to focus light emitted by a point onto an image, even through a flat lens, provided that certain conditions for AANR are met, such as convex isofrequency contours shrinking with frequency about a point in the Brillouin zone [12]. In figure 8, we show such an effect for a perfectly conducting PC in figure 8*b*, and we supplement it, in figure 8*a*, with an endoscope effect using the zero group velocity (or ultra-refraction) effect near *X* along the Brillouin zone, as shown in figure 8*c*. In order to achieve AANR, we choose a frequency on the first dispersion curve (acoustic band) in figure 3, and we take its intersection with the light line *Ω*=|***κ***| along the *XΓ* path. This means that we achieve negative group velocity for waves propagating along the *XΓ* direction of the array, hence the rotation by an angle *π*/4 of every cell within the PC in figure 8*b*. This is a standard trick in optics that has the effect of moving the origin of the light line dispersion to *X* as, relative to the PC, the Bloch wavenumber is along *XΓ*. This then creates optical effects due to the interaction of the light line with the acoustic branch, and this would be absent if *Γ* were the light-line origin. The frequency at which AANR occurs (*Ω*=1.125) is well predicted by the light-line intercept with the classical model, which is the dotted line in figure 8*c*. The effective medium behaves in a hyperbolic fashion since equation (2.11)'s coefficients near that frequency are opposite in sign, *T*_11_=−1.3589 and *T*_22_=0.8725 (with *T*_12_=*T*_21_=0, which is the case throughout the examples). This effective anisotropy is expected and necessary as discussed in Luo *et al.* [12]. Ultra-refraction, figures 8*a* and 9*b*, occurs when one chooses a frequency near a maximum or minimum in the dispersion curve, thereby creating a very slow effective medium within the PC relative to the outer medium, the upshot being that one can create plane-wave emission from a PC slab excited with a line source within the PC. Using equation (3.1) by differentiating with respect to *κ*_1_ and *κ*_2_ and the source's frequency, we can compute the group velocity at both directions as *Ω*,_*κ*_1__=*Ω*,_*κ*_2__=−0.2254.

### (b) Line defect, concentration and endoscope effects in metamaterials

For SRRs with two holes (figure 9), a line defect effect is achieved for a time-harmonic source at a frequency corresponding to the first flat dispersion curve along the *XM* segment of figure 5*a*. Equation (2.11) represents the effective medium, and we can predict this line defect effect since at point *M* of the Brillouin zone, the coefficients *T*_11_=−0.2319 and *T*_22_=0 result in a strongly anisotropic effective medium allowing waves to propagate only in the *x*-direction, which is the vertical direction in figure 5*a*.

An ultra-refraction effect is achieved in figure 9*b* for a frequency corresponding to the first zero group velocity at *Γ* point in figure 5*a*. Near the standing wave frequency for the second mode at point *Γ*, the effective medium governed by equation (2.11) with coefficients *T*_11_=0.2505 and *T*_22_=0.1265 is again anisotropic, and this is due to the asymmetry of the cell in one direction. Using equation (3.1), we differentiate *Ω* with respect to *κ*_1_ and *κ*_2_ for values of *κ*_*i*_=0.2131 that yield a frequency of *Ω*=0.78, and we obtain a group velocity in each of the directions of *Ω*,_*κ*_1__=0.0648 and *Ω*,_*κ*_2__=0.0327, which is extremely small compared to the outside medium's group velocity of *V*_*g*_=1.

A partial lensing effect, light concentration resembling a photonic jet [47], is obtained in figure 9*c* when the frequency of the source is tuned to the value of 1.2 where the region of the second dispersion curve displays a negative group velocity along the *ΓX* direction (hence, the rotation of the array through an angle *π*/4). At point *M*, equation (2.11) has opposite sign coefficients, namely *T*_11_=−1.567 and *T*_22_=0.7707, which give the *f*_0_ equation hyperbolic behaviour and lead to effective anisotropy with light directed along the characteristics. Owing to the asymmetry of the cell, the characteristics are not perpendicular, and yield an image that is slightly shifted with respect to the source.

### (c) Cloaking in metamaterials

**Figure 10.**
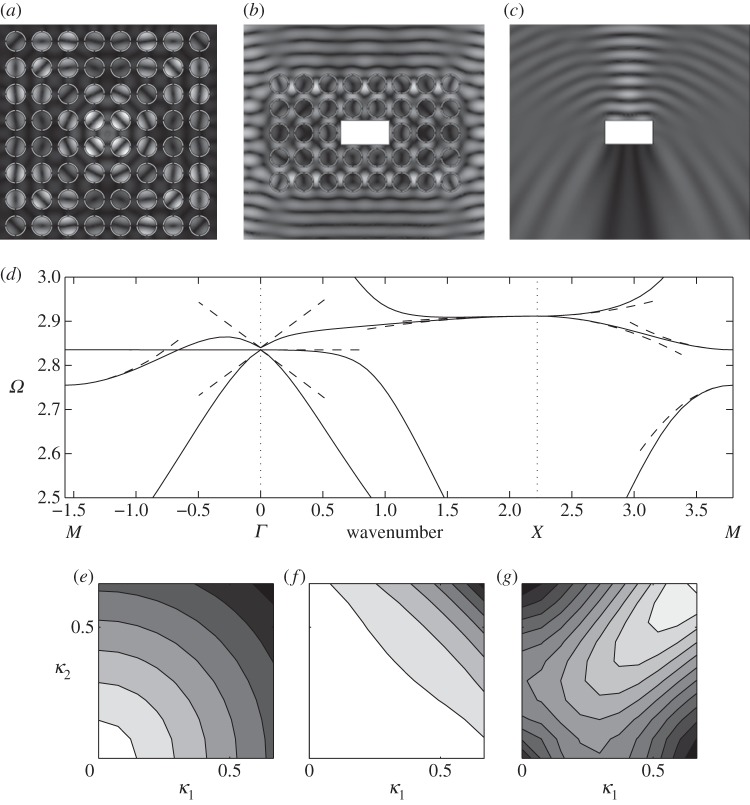
Cloaking in square arrays of SRRs with four holes: a source at frequency *Ω*=2.8, located in the centre of a square metamaterial consisting of 64 SRRs shaped as in figure 5*b* produces a wave pattern reminiscent of (*a*) concentric spherical field, (*b*) cloaking of a rectangular inclusion inside a slab of a metamaterial consisting of 38 SRRs and (*c*) scattering of a plane wave from the same rectangular hole as the previous panel. (*d*) Zoom in dispersion diagram of figure 5*b*. (*e*–*g*) Present isofrequency plots of the lower, middle and upper modes of the Dirac point, respectively.

We now move to SRRs with four holes (figure 10). Clearly, the Mie resonance has faded away in figure 5*b* compared with figure 5*a*, so one should seek other effects than negative refraction. However, the flat band along the *MΓ* path and multiple crossing (Dirac point) shown in figure 10 are interesting. In figure 10*a*, we set a harmonic line source at the corresponding frequency *Ω*=2.8 in an 8×8 array of SRRs and observe a wave pattern of concentric cylindrical modes that are due to the near isotropy of the effective medium at that frequency (cf. the nearly circular isofrequency contours of figure 10*e*). In figure 10*b*,*c*, we show the cloaking of a rectangular defect placed within an array of SRRs. The HFH approach here then acts to shed light upon recent computations by Chan *et al.* [48] that show cloaking in a related context. As can be seen in figure 10*b*, a plane wave propagating at frequency *Ω*=2.8 demonstrates perfect transmission through a slab composed of 38 SRRs; this is because the linear dispersion curves just below the Dirac point are identical to the folded light line of the exterior medium at this frequency. This panel also shows cloaking of a rectangular inclusion where remarkably no scattering is seen before or after the metamaterial slab: figure 10*c* shows the scattering in the absence of the cloak. Figure 10*d* shows the location in the band structure that is responsible for this effect. Note that the frequency of excitation is near, but just below, the Dirac cone point located at *Ω*=2.835 where the group velocity is negative, but also constant near that location of the Brillouin zone, as illustrated through an isofrequency plot of the lower mode of the Dirac point in figure 10*e*. Indeed, the locally isotropic features of figure 10*e* contrast with those of figure 10 *f*,*g*, wherein ultra-flattened isofrequency contours display the hallmarks of ultra-refraction, a regime more prone to omni-directivity than cloaking. The asymptotic system of equations (2.15) describing the effective medium at the Dirac point can be uncoupled to yield the same governing equation for all three 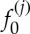
's, such that
5.1
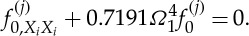

### (d) Lensing via all-angle negative refraction and St Andrew's cross in metamaterials

**Figure 11.**
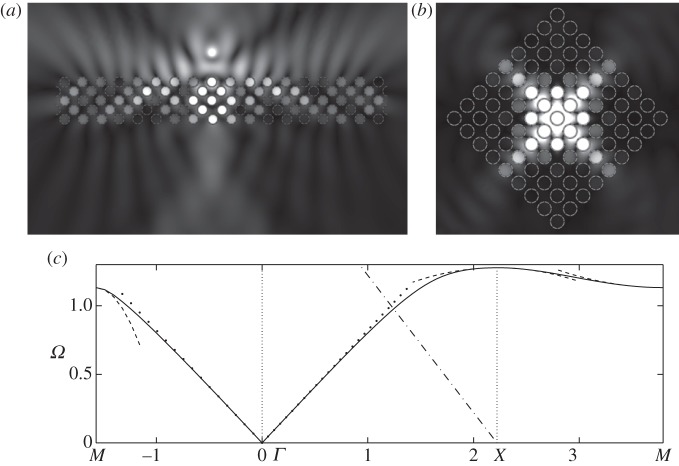
Lensing via AANR and St Andrew's cross in square arrays of SRRs with eight holes: (*a*) a line source at frequency *Ω*=1.1375 located above a rectangular metamaterial consisting of 90 SRRs as in figure 5*c* displays an image underneath (lensing); (*b*) a line source at frequency *Ω*=1.25 located inside a square metamaterial consisting of 49 SRRs as in figure 5*c* displays the dynamically induced anisotropy of the effective medium; (*c*) zoom in dispersion diagram of figure 5*c*. Note that each cell in the arrays in (*a*) and (*b*) has been rotated through an angle *π*/4.

Finally, we demonstrate AANR effects in metamaterials with SRRs with eight holes. The dispersion curves in figure 5*c* are interesting, as the second curve displays the hallmark of AANR of an optical band for a PC (it has a negative group velocity around the *Γ* point). However, this band is the upper edge of a low-frequency stop band induced by the resonance of an SRR, whereas the optical band of a PC results from multiple scattering, which thus arises at higher frequencies. We therefore have a periodic structure behaving somewhat as a composite intermediate between a metamaterial and a PC. We achieve AANR in a way similar to the circular inclusions in figure 8. However, we note that the focusing effect is more pronounced here; the image is much less elongated in figure 11 than in figure 8 and hence has better resolution. Again, the strong anisotropy of the effective material is obvious from coefficients *T*_11_=−5.53 and *T*_22_=0.2946. The same frequency of the first band is obtained at the point *N* of the Brillouin zone, by symmetry of the crystal, we would have *T*_11_=0.2946 and *T*_22_=−5.53. The resultant propagating waves come from the superposition of the two effective media described above. Figure 11*b* further illustrates this anisotropy as the source wave propagates along the predicted directions.

## 6. Concluding remarks

We show conclusively that homogenization theory, and more precisely HFH, captures the essential details of complex geometries within a continuum setting for TE polarization, and the asymptotics that emerge are more accurate and versatile than those of network models. Additionally, the quasi-static low-frequency version of HFH reproduces the classical formulae, and numerically we verify that homogenization theory works in this limit, contrary to statements otherwise.

The examples of cylinders and SRRs are used not merely to verify the theory, but also illustrate how the HFH asymptotics and dispersion curves show unexpected results: the cross-like standing wave patterns of figure 11 are particularly striking. These arise due to strong anisotropy as found from the asymptotics of the dispersion diagram. It is also interesting to note how the resonances in the SRRs change with the inclusion radius, and more importantly, with the number of ligaments whose increase removes the essential resonance that is required for artificial magnetism. Other effects, such as lensing, AANR, ultra-refraction and Dirac cone cloaking, all emerge by choosing critical frequencies that are guided by the asymptotics for the dispersion curves that are accurately found asymptotically using HFH. In the other light polarization case (TM), the structure of the band diagrams changes dramatically, as setting Dirichlet boundary conditions on SRRs (clamped holes in the elasticity) leads to a zero-frequency stop band, whereby classical homogenization breaks down, but not HFH. However, this is beyond the scope of the present paper.

## References

[1] John S 1987 Strong localization of photons in certain disordered dielectric superlattices. Phys. Rev. Lett. 58, 2486–2489 10.1103/PhysRevLett.58.2486 (doi:10.1103/PhysRevLett.58.2486) 10034761

[2] Yablonovitch E 1987 Inhibited spontaneous emission in solid-state physics and electronics. Phys. Rev. Lett. 58, 2059–2062 10.1103/PhysRevLett.58.2059 (doi:10.1103/PhysRevLett.58.2059) 10034639

[3] O’Brien S Pendry JB 2002 Photonic band gap effects and magnetic activity in dielectric composites. J. Phys. Cond. Mat. 14, 4035–4044 10.1088/0953-8984/14/15/317 (doi:10.1088/0953-8984/14/15/317)

[4] Craster RV Guenneau S. eds 2012 Acoustic metamaterials. London, UK: Springer

[5] Torrent D Sánchez-Dehesa J 2008 Anisotropic mass density by two-dimensional acoustic meta-materials. New J. Phys. 9, 023004 10.1088/1367-2630/10/2/023004 (doi:10.1088/1367-2630/10/2/023004)

[6] Joannopoulos JD Johnson SG Winn JN Meade RD 2008 Photonic crystals, molding the flow of light, 2nd edn. Princeton, NJ: Princeton University Press

[7] Pendry JB Holden AJ Stewart WJ Youngs I 1999 Magnetism from conductors and enhanced nonlinear phenomena. IEEE Trans. Microw. Theory Tech. 47, 2075–2084 10.1109/22.798002 (doi:10.1109/22.798002)

[8] Bensoussan A Lions J Papanicolaou G 1978 Asymptotic analysis for periodic structures. Amsterdam, The Netherlands: North-Holland

[9] Jikov V Kozlov S Oleinik O 1994 Homogenization of differential operators and integral functionals. Berlin, Germany: Springer

[10] Mei CC Auriault J-L Ng C-O 1996 Some applications of the homogenization theory. Adv. Appl. Mech. 32, 278–345 10.1016/S0065-2156(08)70078-4 (doi:10.1016/S0065-2156(08)70078-4)

[11] Milton GW 2002 The theory of composites. Cambridge, UK: Cambridge University Press

[12] Luo C Johnson SG Joannopoulos JD 2002 All-angle negative refraction without negative effective index. Phys. Rev. B 65, 201104 10.1103/PhysRevB.65.201104 (doi:10.1103/PhysRevB.65.201104)

[13] Zengerle R 1987 Light propagation in singly and doubly periodic waveguides. J. Mod. Opt. 34, 1589–1617 10.1080/09500348714551531 (doi:10.1080/09500348714551531)

[14] Dowling JP Bowden CM 1994 Anomalous index of refraction in photonic bandgap materials. J. Mod. Optics 41, 345–351 10.1080/09500349414550371 (doi:10.1080/09500349414550371)

[15] Craster RV Kaplunov J Pichugin AV 2010 High frequency homogenization for periodic media. Proc. R. Soc. A 466, 2341–2362 10.1098/rspa.2009.0612 (doi:10.1098/rspa.2009.0612)

[16] Craster RV Kaplunov J Nolde E Guenneau S 2011 High frequency homogenization for checkerboard structures:defect modes, ultra-refraction and all-angle-negative refraction. J. Opt. Soc. Am. A 28, 1032–1041 10.1364/JOSAA.28.001032 (doi:10.1364/JOSAA.28.001032) 21643388

[17] Allaire G Piatnitski A 2005 Homogenisation of the Schrödinger equation and effective mass theorems. Commun. Math. Phys. 258, 1–22 10.1007/s00220-005-1329-2 (doi:10.1007/s00220-005-1329-2)

[18] Birman MS Suslina TA 2006 Homogenization of a multidimensional periodic elliptic operator in a neighborhood of the edge of an internal gap. J. Math. Sci. 136, 3682–3690 10.1007/s10958-006-0192-9 (doi:10.1007/s10958-006-0192-9)

[19] Hoefer MA Weinstein MI 2011 Defect modes and homogenization of periodic Schrödinger operators. SIAM J. Math. Anal. 43, 971–996 10.1137/100807302 (doi:10.1137/100807302)

[20] Nemat-Nasser S Willis JR Srivastava A Amirkhizi AV 2011 Homogenization of periodic elastic composites and locally resonant sonic materials. Phys. Rev. B 83, 104103 10.1103/PhysRevB.83.104103 (doi:10.1103/PhysRevB.83.104103)

[21] Zolla F Renversez G Nicolet A Kuhlmey B Guenneau S Felbacq D 2005 Foundations of photonic crystal fibres. London, UK: Imperial College Press

[22] Ramakrishna SA 2005 Physics of negative refractive index materials. Rep. Prog. Phys. 68, 449–521 10.1088/0034-4885/68/2/R06 (doi:10.1088/0034-4885/68/2/R06)

[23] McPhedran RC Nicorovici NA 1997 The TEM mode and homogenization of doubly periodic structures. J. Elect. Waves Appl. 11, 981–1012 10.1163/156939397X00378 (doi:10.1163/156939397X00378)

[24] Nicorovici NA McPhedran RC Botten LC 1995 Photonic band gaps:non-commuting limits and the acoustic band. Phys. Rev. Lett. 75, 1507–1510 10.1103/PhysRevLett.75.1507 (doi:10.1103/PhysRevLett.75.1507) 10060315

[25] Poulton CG Botten LC McPhedran RC Nicorovici NA Movchan AB 2001 Non-commuting limits in electromagnetic scattering: asymptotic analysis for an array of highly conducting inclusions. SIAM J. Appl. Math. 61, 1706–1730 10.1137/S0036139999352262 (doi:10.1137/S0036139999352262)

[26] Halevi P Krokhin AA Arriaga J 2001 Comment on Photonic band gaps:noncommuting limits and the acoustic band. Phys. Rev. Lett. 86, 3211–3211 10.1103/PhysRevLett.86.3211 (doi:10.1103/PhysRevLett.86.3211) 11290150

[27] Nicorovici NA McPhedran RC Botten LC 2001 Comment on ‘Photonic band gaps: noncommuting limits and the ‘acoustic band’ ’—Nicorovici, McPhedran, and Botten reply. Phys. Rev. Lett. 86, 3212–3212 10.1103/PhysRevLett.86.3212 (doi:10.1103/PhysRevLett.86.3212) 10060315

[28] Mei CC Vernescu B 2010 Homogenisation methods for multiscale mechanics. Singapore: World-Scientific Publishing

[29] Parnell WJ Abrahams ID 2006 Dynamic homogenization in periodic fibre reinforced media:quasi-static limit for SH waves. Wave Motion 43, 474–498 10.1016/j.wavemoti.2006.03.003 (doi:10.1016/j.wavemoti.2006.03.003)

[30] McIver P 2007 Approximations to wave propagation through doubly-periodic arrays of scatterers. Waves Random Complex Media 17, 439–453 10.1080/17455030701481831 (doi:10.1080/17455030701481831)

[31] Li Y Mei CC 2007 Multiple resonant scattering of water waves by a two-dimensional array of vertical cylinders:linear aspects. Phys. Rev. E 76, 016302 10.1103/PhysRevE.76.016302 (doi:10.1103/PhysRevE.76.016302) 17677558

[32] Brillouin L 1953 Wave propagation in periodic structures:electric filters and crystal lattices, 2nd edn. New York, NY: Dover

[33] Craster RV Antonakakis T Makwana M Guenneau S 2012 Dangers of using the edges of the Brillouin zone. Phys. Rev. B 86, 115130 10.1103/PhysRevB.86.115130 (doi:10.1103/PhysRevB.86.115130)

[34] Craster RV Kaplunov J Postnova J 2010 High frequency asymptotics, homogenization and localization for lattices. Q. J. Mech. Appl. Math. 63, 497–519 10.1093/qjmam/hbq015 (doi:10.1093/qjmam/hbq015)

[35] Nolde E Craster RV Kaplunov J 2011 High frequency homogenization for structural mechanics. J. Mech. Phys. Solids 59, 651–671 10.1016/j.jmps.2010.12.004 (doi:10.1016/j.jmps.2010.12.004)

[36] COMSOL. 2012 COMSOL Multiphysics. See http://www.comsol.com

[37] Guenneau S Zolla F 2000 Homogenization of three-dimensional finite photonic crystals. Progr. Electromagn. Res. 27, 91–127 10.2528/PIER99071201 (doi:10.2528/PIER99071201)

[38] Villeneuve PR Piché M 1992 Photonic band gaps in two-dimensional square and hexagonal lattices. Phys. Rev. B 46, 4969–4972 10.1103/PhysRevB.46.4969 (doi:10.1103/PhysRevB.46.4969) 10004259

[39] Martinsson PG Movchan AB 2003 Vibrations of lattice structures and phononic band gaps. Q. J. Mech. Appl. Math. 56, 45–64 10.1093/qjmam/56.1.45 (doi:10.1093/qjmam/56.1.45)

[40] Ayzenberg-Stepanenko MV Slepyan LI 2008 Resonant-frequency primitive waveforms and star waves in lattices. J. Sound Vib. 313, 812–821 10.1016/j.jsv.2007.11.047 (doi:10.1016/j.jsv.2007.11.047)

[41] Colquitt DJ Jones IS Movchan NV Movchan AB McPhedran RC 2012 Dynamic anisotropy and localization in elastic lattice systems. Waves Random Complex Media 22, 143–159 10.1080/17455030.2011.633940 (doi:10.1080/17455030.2011.633940)

[42] Osharovich G Ayzenberg-Stepanenko M Tsareva O 2010 Wave propagation in elastic lattices subjected to a local harmonic loading. II. Two dimensional problems. Contin. Mech. Thermodyn. 22, 599–616 10.1007/s00161-010-0164-7 (doi:10.1007/s00161-010-0164-7)

[43] Guenneau S Movchan AB Petursson G Ramakrishna SA 2007 Acoustic metamaterials for sound focusing and confinement. New J. Phys. 9, 399 10.1088/1367-2630/9/11/399 (doi:10.1088/1367-2630/9/11/399)

[44] Llewellyn-Smith SG Davis AMJ 2010 The split ring resonator. Proc. R. Soc. A 466, 3117–3134 10.1098/rspa.2010.0047 (doi:10.1098/rspa.2010.0047)

[45] Movchan AB Guenneau S 2004 Split-ring resonators and localized modes. Phys. Rev. B 70, 125116 10.1103/PhysRevB.70.125116 (doi:10.1103/PhysRevB.70.125116) 19905438

[46] Kozlov V Maz’ya V Movchan AB 1999 Fields in multistructures:asymptotic analysis. Oxford, UK: Oxford University Press

[47] Ferrand P Wenger J Devilez A Pianta M Stout B Bonod N Popov E Rigneault H 2008 Direct imaging of photonic nanojets. Opt. Express 16, 6930–6940 10.1364/OE.16.006930 (doi:10.1364/OE.16.006930) 18545397

[48] Chan CT Huang X Liu F Hang ZH 2012 Dirac dispersion and zero-index in two dimensional and three dimensional photonic and phononic systems. Progr. Electromagn. Res. B 44, 163–190 10.2528/PIERB12082103 (doi:10.2528/PIERB12082103)

